# Strategic filtering of high-energy visible light expands neural correlates of functional vision particularly in older participants

**DOI:** 10.1016/j.heliyon.2023.e17271

**Published:** 2023-07-05

**Authors:** Billy Hammond, Marissa Gogniat, John Buch, L. Stephen Miller

**Affiliations:** a Vision Sciences Laboratory, Behavioral and Brain Sciences Program, Department of Psychology, University of Georgia, Athens, GA, USA; b Vanderbilt Memory & Alzheimer's Center, Vanderbilt University Medical Center, Nashville, TN, USA; c Research & Development, Johnson & Johnson Vision Care Inc., Jacksonville, FL, USA; d Neuropsychology and Memory Assessment Laboratory, Department of Psychology, University of Georgia, USA

**Keywords:** Functional MRI, BOLD response, Glare disability, Filtering

## Abstract

In this study we assessed the neural correlates of functional vision while varying patterns of light filtration. Four filter conditions used relatively flat filtering across the visible spectrum while one filter was a step filter that selectively absorbed violet light (wavelengths below about 415 nm). Neural effects were quantified by measuring the BOLD response ((T2*-based fMRI) while subjects performed a challenging visual task (judging gap direction in Landolt Cs that randomly varied in size). In general (based on p < 0.01 directional criterion not corrected for aggregated error), as filtering increased (less interference by bright light), brain activity associated with the task also increased. This effect, even using the most conservative statistics, was most evident when using the violet filter (especially for the older subjects) despite only reducing the very highest energy portion of the visible spectrum. This finding suggests that filtering can increase neural activity associated with functional vision; such effects might be achievable through filtering just the highest visible energy (violet).

## 1 Introduction

One of the more impressive features of the visual system is its ability to operate over an extreme range of stimulus energies. The system can derive a useable signal despite ambient light levels that can vary abruptly and over an immense range. This capability is based on numerous (often non-exclusive) features but, notably, the ability to vary sensitivity and behavioral strategies. For example, short-wave (blue-violet) light, which expands the range of color vision but may have other deleterious optical features (such as being subject to higher atmospheric scatter, increased chromatic aberration and photo-oxidative potential) is filtered by the crystalline lens and the macular pigments. The visual system, however, can compensate for this filtering even when the combined optical density exceeds one log unit (<5% transmission at 460 nm) [1,2]. The incoming light signal can be further modified behaviorally through the use of tinted spectacle lenses, contact lenses, and intraocular implants. One design goal of such lenses is to reduce the glaring effects of bright light [3] while simultaneously minimizing the impact of filtering on other visual and non-visual (e.g., circadian rhythms) processes [4,5]. For example, intrinsically photosensitive retinal ganglion cells are maximally sensitive around 480 nm so many intraocular implants that are “blue-blocking” are actually more relegated to the violet [6]. A major challenge in this area is designing filters that work optimally and synergistically with these very complex and dynamic systems.

Using neuroimaging responses to study light stress has been done in previous research on populations such as photophobic individuals with migraines [7,8]. If a light looks different, say brighter, that means by definition it has provoked a different underlying neural response. The question has been whether current neuroimaging methods have the sensitivity to measure that response and with what specificity (e.g., at the simplest level, a brighter light should reflect higher overall activation of occipital areas [9]). Past work has shown that the trigeminal nerve (cranial nerve V) is closely involved with the perception of visual discomfort due to excessive light (possibly exacerbated by the short-wave component [10]). Huang et al. (2011) specifically studied [11] the effect of light filtering on participants with photosensitive migraines. Huang et al. concluded that precision spectral filters can normalize activity both in the extra-striate visual areas and overall cortical activation to the degree that migraineurs perform similarly to non-headache control participants. This finding made sense: bright light increases visual stress and activation of the brain in some areas, it also, however, if sufficiently bright, has the ultimate effect of inactivating visual function (i.e., once cannot see, hence the term disability glare).

Although there has been considerable neuroimaging work done studying visual discomfort in these type of clinical cases (e.g., migraine, glaucoma, reading disorder, Scotopic Sensitivity Syndrome [11]), there is relatively little neuroimaging work on the use of filtering to expand useable vision in normal healthy subjects (most neuroimaging work on normal subjects has focused on visual fatigue induced by stereoscopy [12]). A clue certainly can be based on natural intraocular filters which tend to focus on the high-energy short-wave end of the visible spectrum. Filters of various types (tinted windows, spectacles, contact lenses, intraocular implants) have long been used to expand visual dynamic range [13]. In this study, we tested several filtering designs to see how visual function (and the underlying neural response) was affected when performing a visual task under bright “natural” light (a xenon source was used to emulate sunlight).

The study design was based on several assumptions:

1. A challenging visual-motor task (judging the gap direction in Landolt Cs that varied, randomly, in size) would activate significant areas of brain measurable with fMRI [9].
2. By adding light stress, the task would be even more challenging (more aversive light would make the visual targets harder to see). As the task was “disabled,” less brain activity would be evoked.
3. Extrinsic filters, by attenuating light stress and reducing disability, would influence this effect. We hypothesized that the highest brain activity would be related to the filter that most facilitated task performance.

**Fig. 1:**
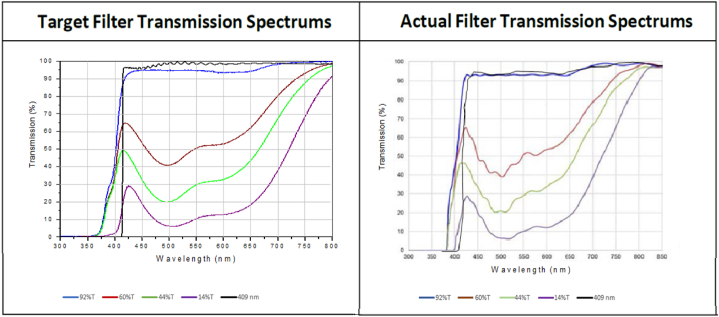
Filters used include 92% transmission (blue), 60% transmission (red), 44% transmission (green), 14% transmission (purple) and the 409 step filter (black). The panel on the left shows the spectra as specified by the manufacturer (Semrock). The spectra on the right show the filters as measured (ILT950 spectroradiometer) at the plane of the subject's eye using the filters in place and the xenon light source. (For interpretation of the references to color in this figure legend, the reader is referred to the Web version of this article.)

**Fig. 2:**
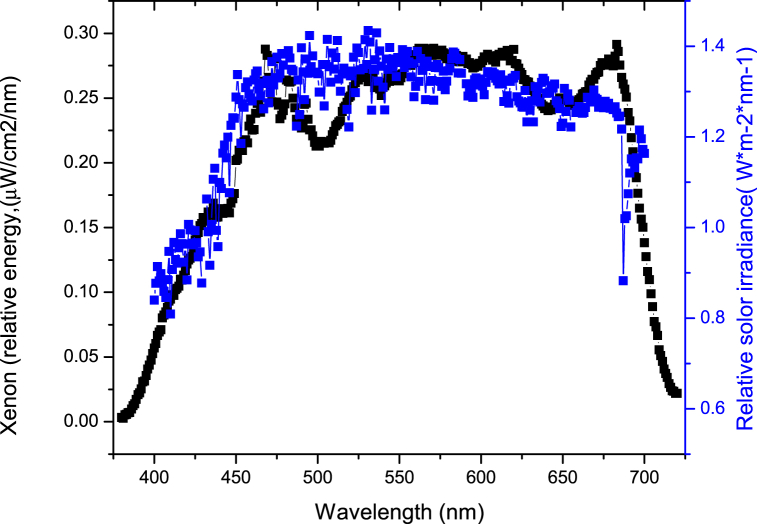
Black trace: the unattenuated emission spectrum of the xenon light source (measured at the plane of the eye) that was used in the no-filter condition. Blue trace: Spectrum of daytime sunlight from G173-03e1 Standard Tables for Reference Solar Spectral Irradiances: Direct Normal and Hemispherical on 37° Tilted Surface, copyright ASTM International (ASTM), 100 Barr Harbor Drive, West Conshohocken, PA 19428. A copy of the complete standards may be obtained from ASTM, www.astm.org. (For interpretation of the references to color in this figure legend, the reader is referred to the Web version of this article.)

Per our hypothesis, we included a sharp step filter that cut out only violet light. Past studies have suggested a strong aversive effect of very short-wavelength light [14]. The filtering conditions we used are shown in Fig. 1. The no filter condition (compared to the solar spectrum), showing the xenon light source that was used, is shown in Fig. 2 (neural activation in this no filter condition served as the baseline for the fMRI analysis).

**Fig. 3:**
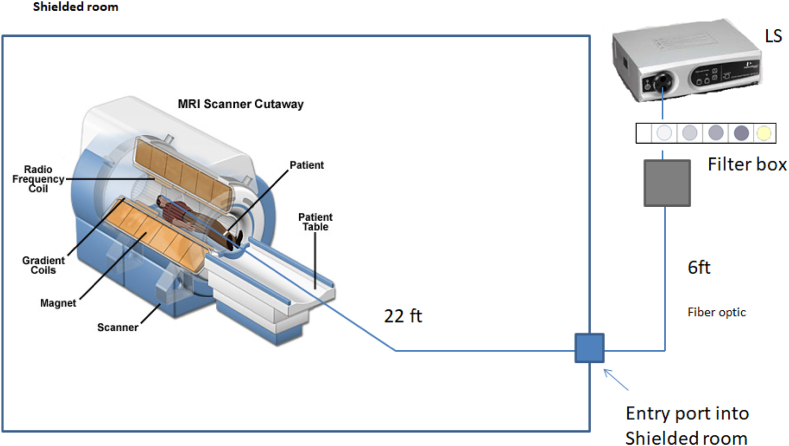
Schematic of the testing conditions.

As shown in the figures, four of the filters that were used had similar attenuation profiles but varied the amount of light filtered. The fifth (the -violet filter) was a strong step filter that sharply cut off just the very edge of the visual spectrum (where visual sensitivity is very low). We also assessed visual comfort, subjectively and objectively, while the subjects performed the visual task. For the objective assessment, we conducted a whole brain analysis within the fMRI device. It is well-known that the entire brain is engaged when conducting visually/cognitively challenging tasks [15]but we also focused on areas known to process visual stimuli. The experimental setup (shown in Fig. 3) allowed us to assess the effects of varying light stress (both in quantity and type) on the neural output accompanying performance of that task.

## 2 Methods

### 2.1 Subjects

The participants were 24 healthy males (15) and females (9) with uncorrected, broadly normal vision from the community; a young adult group (twelve 18–28 y/o) and an older group (twelve 60–70 y/o). All participants had uncorrected visual acuity of 20/70 or better in each eye. Standard exclusion criteria were applied including incompatibility with the MRI (e.g., ocular disease, pregnancy, metal implants, etc.) but no subjects needed to be excluded on this basis. All study procedures and materials were approved by the University of Georgia Institutional Review Board prior to initiating the study. All participants gave both written and verbal informed consent prior to participation. The tenets of the Declaration of Helsinki, and the International Conference on Harmonization Good Clinical Practice E6 (ICH-GCP) were adhered to at all times while conduction the study.

### 2.2 General study design

This was a 2-visit, subject-masked, 6 treatment (filter) by 6 period (6 × 6) crossover study. The participants had no prior knowledge of specific illuminance/filter/target combinations that they experienced. There were 5 filter conditions (4 based on % T suppression; 1 based on wavelength filtering) and a no-filtering control (presented in randomized order). The filters were supplied by Semrock, Rochester, NY. The radiant output for the no-filtering lens condition is shown in Fig. 2 (equivalent to 2280 Lux). The photometric output for the 92, 60, 44 and 14% transmission filters were 2120, 1160, 678, 255 Lux, respectively. The violet filter ing condition was 2250 Lux.

Participants were briefly exposed to a no-filter condition interposed between each presentation (at an illuminance level of about 2280 Lux). Cortical activity was measured with T2*-based functional magnetic resonance imaging (fMRI). Participants were recruited for and stratified into one of two age groups using a 1:1 allocation ratio.

The general testing arrangement is schematized in Fig. 3. As shown, the 300-Watt xenon light source (Cermax, Excelitas Technologies, Fremont, CA) was located outside the room and provided the light we used to emulate sunlight (CCT = ∼6000K, x = 0.32, y = 0.325 calibrated with the ILT950 spectroradiometer [16]). Light output from this system was checked periodically throughout the experiment to ensure that the output remained stable. Light from the xenon system was carried into the bore of the MRI through a specially made high-fidelity fiber optics cable (Atlas Specialty Lighting) that bifurcated to deliver the light to each side of the subject. The ends of the light guides were incorporated into the head stabilization assembly such that the light was delivered to each side of the subjects’ field of view (bracketing the visual image). This light guide was interrupted (outside of the shielded test room) with a custom designed filter box that allowed the easy interposition of the various filters we were testing. Participants were exposed to the different filtering conditions while performing an active visual task (judging the gap direction of Landolt Cs).

### 2.3 Neuroimaging procedures

The General Electric (GE) 3 T HDx magnetic resonance system (Waukesha, WI) in the University of Georgia's Bioimaging Research Center was utilized for all neuroimaging acquisitions. Structural scans were collected using a high-resolution three-dimensional T1-weighted fast-spoiled gradient recall echo sequence [repetition time (TR) = 7.5 ms; echo time (TE) = <5 ms; field of view (FOV) = 256 × 256 mm matrix; flip angle = 20°; slice thickness = 1 mm; 154 axial slices; voxel size = 0.94 × 0.94 × 1 mm] with a total acquisition time of 6 min, 20 s. This protocol covered the top of the head to the brainstem and collected 176 images. Functional scans were aligned to the anterior commissure/posterior commissure line and collected axially using a T2*-weighted single shot echo planar imaging (EPI) sequence (TR = 1500 ms; TE = 25 ms; 90° RF pulse; acquisition matrix = 64 × 64; FOV = 220 × 220 mm; in-plane resolution, 220 × 64 mm; slice thickness = 4 mm; 30 interleaved axial slices; voxel size = 3.43 × 3.43 × 4 mm) with an acquisition time of 12min, 24 s. The EPI sequence covered the cortical surface and a portion of the cerebellum, and consisted of 290 volumes. A pair of magnitude and phase images was acquired, lasting 1 min, 40 s each, for field map-based unwarping (TR = 700 ms; TE = 5.0/7.2 ms; FOV = 220 × 220 mm matrix; flip angle = 30°; slice thickness = 2 mm; 60 interleaved slices; voxel size = 1.72 × 1.72 × 2 mm). Imaging data were processed and analyzed using Statistical Parametric Mapping (SPM12, Welcome Department of Cognitive Neurology, London, UK). For pre-processing, images were first converted from GE DICOM format to NIFTI format using the dcm2nii conversion tool [17]. Each subject's data was slice time corrected to account for the interleaved acquisition. Data then underwent realignment and unwarping procedures to adjust for any distortion that may have resulted from magnetic field inhomogeneities and movement during the scan. The anatomical scan for each participant was co-registered to the functional images through a transformation process, and then segmented into gray matter, white matter, and cerebrospinal fluid, which aids the normalization of functional images into normal space. Functional images were then normalized to the Montreal Neurological Institute (MNI) template using a nonlinear, 12-parameter affine transformation registration, and smoothed with a 6.75-mm FWHM Gaussian filter to de-emphasize random noise and increase the signal-to-noise ratio. Following pre-processing, 1st level analyses was used to identify the conditions and timing of the paradigm. A Matlab file (i.e., .mat) was used to identify the onset of the blocks in seconds.

Region of Interest (ROI) analyses [18] were performed for the illumination contrasts and for the filtering contrasts separately and together using the cluster level inference method (statistical threshold p < 0.005, 8 contiguous voxels). ROIs were identified *a priori* based on previous literature on visual functioning patterns to illumination and included the bilateral occipital lobes, visual areas V1–V5 combined, and V1 – V5 individually. Similarly, initial whole brain analyses were included as a summary technique for overall activation and location identification.

### 2.4 Subjective responses

The testing paradigm consisted of being flashed a bright light for 90 s, during which time the subjects attempted to correctly identify the gap in the randomly-oriented Landolt Cs.

Once the 90 s of light was over, the subjects had 3 s to answer Question (Q) 1, then 3 s to answer Q 2, and then 3 s to answer Q 3. They were then allowed 2 min of rest. This was repeated twice per filter condition for all six filter conditions. Subjects needed to remain relatively motionless (≤1 mm) in the bore of the MRI for about 1 h and 15 min.

The questions were: (Q1) “How Comfortable Was the Light?” (Range, 0 Extremely uncomfortable; 3–4 mildly uncomfortable; 7–9 Extremely comfortable); (Q2) “How Much Glare Did You Experience?” (Range, 0 Extreme glare; 3–4 mild glare; 7–9 No glare”; (Q3) “How well could you see the task?” (Range, 0 Not well; 3–4 moderately well; 7–9 Extremely well).

### 2.5 Statistical methods

All statistical tests for the primary analyses were 1-sided (based on a directional hypothesis), conducted at the whole brain level using uncorrected p values of <0.01. We used this more liberal comparison method (i.e., not the more stringent FEW correction method) due to the strong basis for a directional hypothesis, relatively small sample size, and more exploratory methodology.

Image preprocessing was as noted above. Following pre-processing, first level analysis contrasted activation differences between the illumination baseline condition (no suppression filter) and each filter condition for each participant. Second level analysis performed these contrasts at a group level, for all participants (i.e., all participants, older adults, young adults). Where feasible, additional analyses compared the contrasts between the two age groups to determine age effects on visual activation related to illumination and filtering of light. Analyses were performed across the whole brain and for specific regions of interest (ROIs: Occipital lobe; V1; V2; V3; V4; V5, and V1–V5 combined).

## 3 Results

**Fig. 4:**
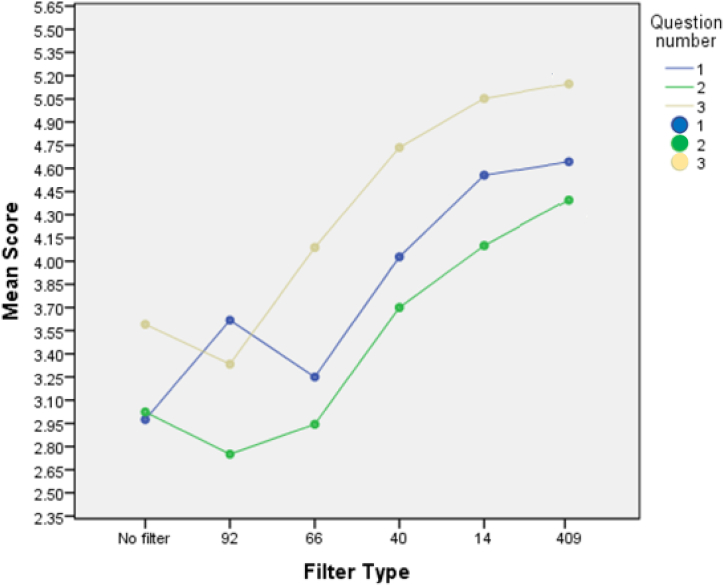
Average responses to the three subjective questions by filter condition (n = 24). Question (Q1) 1: how comfortable was the light? (higher number = more comfortable) Question (Q2) 2: How much glare did you experience ? (higher number = less glare) Question (Q3) 3: How well could you see the task? (higher number = more visible). The standard deviation values for the control lens (100%T) was Q1 (1.98), Q2 (1.71) and Q3 (2.16). The standard deviation values for the 44%T lens was Q1 (1.86), Q2 (1.64) and Q3 (1.67). The standard deviation values for the 409 nm step lens was Q1 (2.23), Q2 (1.81) and Q3 (1.97).

The response to the subjective questions (young and older) is shown in Fig. 4. The differences for the violet filter condition were highly significant (Q1, p < 0.016; Q2, p < 0.019; Q3, p < 0.026). This difference was largely driven by the older participants (the effect for the 12 younger subjects was not significant when considered separately). The results for the remaining questions were trending but not statistically significant.

Using the most conservative statistics (correcting for family wise error (FWE) and using two-tailed hypotheses) and focusing only on the four broad spectrum filters shown in Fig. 1, we found no significant brain effects of the varying filters compared to a non-filtering lens for our total group, nor for young nor older adult groups separately. This was true for both whole brain levels and when looking specifically at *a priori* vision-specific regions of interest (ROI). We suspect that this lack of an effect was likely due to the relatively small sample size (12 younger and 12 older subjects).

**Fig. 5:**
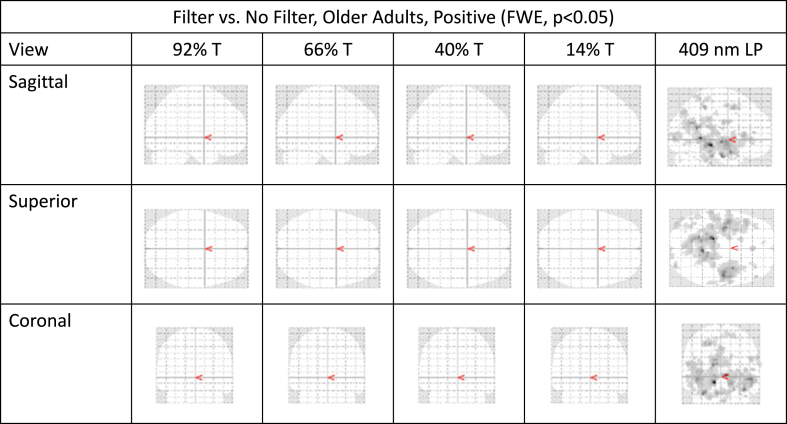
Differences in positive neural activation (statistical parametric mapping, SPM results) when comparing task performance (n = 12, older participants) using no filter, the 44%T filter or the 409 nm step filter. Height threshold T = 9.45, Extent threshold K = 0 voxels. The no filter condition is represented by no gray coloration (“zeroed”), gray shading reflects activation. The 44%T neutral density filter showed mild alteration that was only detectable when using the most liberal statistical criteria. The 409 nm long pass filter enabled significantly more brain activity that was detectable with even the most conservative statistical analysis.

Since we started the study with a directional hypothesis and a relatively small sample, we applied a more liberal thresholding criteria [19]. When this was done, findings at the whole brain level and at the ROI levels suggested differential activation of the filters. An increase in activation in frontal areas of the brain as a roughly dose response in the expected direction was found (driven primarily by the younger subjects). We also evaluated the significantly activated voxels across the whole brain, contrasting full illumination (no filter) to the violet step filter. Using the most conservative approach, we found whole brain effects for the older group only (the step filter significantly changed activation across the brain, primarily positive/increased). These data are shown for whole brain in Table 1 and Fig. 5.

**Table 1:** The difference in positive and negative neural activation (i.e., filtered BOLD response greater or less than the baseline no filter BOLD response) when conducting a visual task in older subjects (n = 12) (409 nm filter vs no filter), FWE <0.05. Filter 409 vs No Filter, Older Adults, Positive (FWE, p < 0.05).

Cluster level (k)	p-value (FWE)	x coordinate (mm)	y coordinate (mm)	z coordinate (mm)	Name (Harvard-Oxford Atlas)
5141	.000	−14	−30	−12	Parahippocampal gyrus
	.000	6	−48	0	Lingual gyrus
	.000	−34	−12	−30	Parahippocampal gyrus
1444	.000	50	−14	−6	Superior temporal gyrus
	.000	38	2	−22	Planum polare
	.000	52	−4	−12	Superior temporal gyrus
59	.000	−22	−40	48	Left cerebral white matter
82	.000	−30	8	50	Middle frontal gyrus
114	.000	−42	−90	−2	Lateral occipital cortex
	.000	−36	−90	6	Occipital pole
	.000	−50	−80	4	Lateral occipital cortex
31	.000	34	−92	2	Occipital pole
209	.000	−60	−34	18	Planum temporale
	.000	−50	−26	10	Heschl's gyrus
	.000	−50	−30	18	Parietal operculum cortex
42	.000	4	34	−2	Cingulate gyrus
56	.000	50	−10	24	Postcentral gyrus
31	.000	60	−2	26	Precentral gyrus
14	.000	52	40	10	Frontal pole
17	.000	32	−14	−24	Parahippocampal gyrus
8	.000	32	−12	−30	Parahippocampal gyrus
14	.000	−16	−4	32	Superior Frontal Gyrus
12	.001	−50	−68	26	Lateral occipital lobe
24	.001	−52	−34	2	Superior temporal gyrus
51	.001	−38	−66	−6	Occipital fusiform gyrus
10	.001	−42	14	−28	Temporal pole
22	.001	−14	16	48	Superior frontal gyrus
85	.001	12	−50	64	Superior parietal lobe
	.029	−2	−54	60	Precuneous cortex
12	.001	−40	−60	24	Angular gyrus
45	.002	20	−38	62	Postcentral gyrus
29	.003	−38	38	8	Frontal pole
43	.003	62	−34	30	Parietal operculum cortex
25	.003	14	−60	26	Precuneous cortex
6	.003	66	−12	16	Postcentral gyrus
19	.004	−38	−74	18	Lateral occipital cortex
2	.005	−50	−58	−30	No label found
40	.006	14	98	6	Occipital pole
	.026	2	98	4	Occipital pole
18	.006	34	38	−10	Frontal pole
	.023	30	32	−4	Frontal orbital cortex
5	.007	−6	52	−6	Frontal medial cortex
16	.008	−2	−22	40	Cingulate gyrus
16	.009	−54	−54	10	Middle temporal gyrus
3	.012	−12	10	40	Paracingulate gyrus
1	.012	68	−16	18	Postcentral gyrus
1	.012	−48	12	−20	Temporal Pole
10	.014	−50	−50	−6	Middle temporal gyrus
4	.015	−12	−28	60	Precentral gyrus
4	.015	−36	−70	−24	Occipital fusiform gyrus
4	.018	40	2	−32	Temporal fusiform cortex
2	.022	40	10	−32	Temporal pole
3	.027	−28	−76	−2	Occipital fusiform gyrus
3	.028	14	82	−30	No label found
2	.031	−50	10	−22	Temporal pole
1	.035	−16	14	−22	Temporal pole
12	.036	60	−26	20	Parietal operculum Cortex
1	.038	14	−82	−38	No label found
3	.04	−50	−60	−8	Inferior temporal gyrus
1	.043	56	22	8	Inferior frontal gyrus
1	.045	−38	−52	−34	No label found
1	.045	−52	−60	32	Lateral occipital cortex
1	.048	42	22	−6	Frontal orbital cortex
1	.048	10	−80	−36	No label found
1	.049	38	−86	−6	Lateral occipital cortex
1	0.49	32	−60	−2	Temporal occipital fusiform cortex

Cluster level (k)	p-value (FWE)	x coordinate (mm)	y coordinate (mm)	z coordinate (mm)	Name (Harvard-Oxford Atlas)
5	.000	−66	−26	34	Supramarginal gyrus
6	.003	28	26	44	Middle frontal gyrus
3	.005	−60	−62	24	Lateral occipital cortex
10	.008	0	18	6	Subcallosal cortex
3	.019	22	24	10	Right cerebral white matter
1	.036	−20	18	12	Left cerebral white matter

Filter 409 vs No Filter, Older Adults, Negative (FWE, p < 0.05).

Using the more liberal threshold that we used to analyze the other filters (uncorrected *p* values of < 0.01), extended the results to include activation differences in the young group as well. Both young and old (and somewhat together) had multiple areas of the brain show positive (increased) activation and negative (decrease) activation relative to the no filter condition. In contrast to the anterior/posterior and positive/negative activation pattern seen in the younger adults’ dose response-like relationship found earlier, these results suggest multiple areas of increased and decreased activation compared to no filter in many brain areas, and in both younger and older groups.

## 4 Discussion

In general, we found relatively modest effects of filtering on the fMRI BOLD response to visual tasks when using the most conservative analysis plan. Since this study had a relatively small sample size (a limitation of this study), however, we applied a more liberal (directional) thresholding approach (without correcting for multiple tests, FWE) and found an approximate dose response between light suppression (from the filters shown in Fig. 1) and cortical activation in occipital areas (coupled with increases in frontal activation): Namely, as light stress decreased due to filtering, brain activation increased (i.e., the brain of the subjects were engaged in performing the task and was not inactivated by the increased glare of the surrounding light). This makes sense: sensory acquisition is the first stage of information processing and task performance. If a glaring light source impairs initial encoding, subsequent processing would also be impaired. Of note, this was seen most clearly in the younger adult group with very little influence on activation changes by light attenuation in the older group (with the exception of the -violet filter as explained below). This likely reflects a larger “swamping” effect of bright light, even partially attenuated, in the older group (several conditions were over 2000 Lux within a single hour session which was bright enough to wash out the task for most of the older subjects).

Of significant interest was the seemingly disproportionate effect of the violet filter. As can be seen in Fig. 1, the effect of this filter on the visible spectrum is minimal. For example, the no filter condition was 2280 Lux and the violet filter condition was 2250 Lux (only 1–2% less in overall illuminance although about 30% less in energy when considering just the 380–415 nm waveband; see Fig. 1). In contrast, the 14% transmitting filter was only 255 Lux. Nonetheless, the subjects responded that they found the violet filter condition similar to the most optically dense filter (only 14% overall transmission but similar to the violet filter in energy reduction between 380 and 415 nm): these were the most comfortable, least glaring, and most amenable to task performance. This subjective response was mirrored by the neuroimaging results. Comparing the BOLD effects of violet filter condition with the no-filter condition yielded significant activation differences. This was seen in the combined groups as well as the older group alone.

Based on our analysis, however, it was clear that the violet filter (despite relatively minimal absorbance of the visible spectrum) made a larger difference for the older subjects compared to the younger subjects. Hence, we were able to pick up the differences in activation for the older subjects even when we used the most conservative level of analysis. In contrast, we were only able to see this effect in the younger adults using a more liberal thresholding technique.

The fact that the violet filter condition yielded the most significant difference in both our subjective and objective analysis suggests that the effect was likely not driven by the brightness or glaring nature of the stimulus per se (see Fig. 1). Why then was there such a large effect of this type of filtering?

One possibility is that there were other meaningful differences in the optical properties of the filters. For example, the more optically dense filters that we used changed the chromatic content of the white light stressor incident at the subject's eye (see right panel Fig. 1). The violet filter (and the 92T), in contrast, did not alter the white appearance of the light source. Since the task involved white Landolt Cs on a black background, a color change could have changed the inherent contrast of the letters (and hence the difficulty of the task, visibility of the letters, etc). Filtering is also often achieved through reflection which can add light scatter exacerbating glare. If the behavioral/brain responses we measured were influenced by optical differences introduced by the actual filtering, it does emphasize that the strategic nature of filtering may be as important as the absolute but undifferentiated quantity of light filtered.

That leads to a related possibility. The very shortest wavelengths are prone to a number of perceptual and optical aberrations that might be expected to influence the kind of task these subjects were required to perform (judging gaps in a Landolt C while being exposed to bright off-axis white light). For example, longitudinal chromatic aberrations at 400–410 nm is over two diopters [20] and shorter wavelengths, even at equal energy, evoke more glare discomfort [14]. Reading and Weale (1974) originally calculated that by filtering short-wave light (by about 14%) the violet penumbra of a white disc would be reduced to threshold levels [21]. By reducing the most visually deleterious (and actinic [22]) portion of the light, the violet filter may be allowing for more processing of the task in specific brain regions than was able to be accomplished under the no filter condition (without significantly altering chromatic content).

## Author contribution statement

Billy Hammond: Stephen Miller: Conceived and designed the experiments; Performed the experiments; Analyzed and interpreted the data; Contributed reagents, materials, analysis tools or data; Wrote the paper.

Marissa Gogniat: Performed the experiments; Analyzed and interpreted the data; Wrote the paper.

John Buch: Conceived and designed the experiments; Analyzed and interpreted the data; Wrote the paper.

## Data availability statement

Data will be made available on request.

## Financial Support

This study was supported by Johnson & Johnson Vision Care Inc.

## Declaration of competing interest

The authors declare the following financial interests/personal relationships which may be considered as potential competing interests: We confirm that Johnson and Johnson Vision funded this research. John Buch is a current employee of Johnson and Johnson Vision and Billy Hammond has consulted for that company in the last three years. Steve Miller and Marissa Gognia have no conflicts to report.
